# The utility of next generation sequencing targeted multigene panels in the Adult Neurogenetic Clinic at Tygerberg Hospital, South Africa

**DOI:** 10.1038/s41431-025-01900-2

**Published:** 2025-06-25

**Authors:** Cumine Van Tonder, Mardelle Schoeman, Jonathan Carr, Franclo Henning, Claude Bailly, Shahida Moosa

**Affiliations:** 1 Division of Molecular Biology and Human Genetics, Faculty of Medicine and Health Sciences, Stellenbosch University, Cape Town, South Africa; 2 Medical Genetics, Tygerberg Hospital, Cape Town, South Africa; 3 Division of Neurology, Department of Medicine, Faculty of Medicine and Health Sciences, Stellenbosch University, Cape Town, South Africa; 4 Genomics for Health in Africa (GHA), Africa-Europe Cluster of Research Excellence (CoRE), Cape Town, South Africa

**Keywords:** Neurological disorders, Genetics research

## Abstract

Next generation sequencing (NGS) based tests have become first-line investigative modalities in adult neurogenetic clinics. Studies in high-income countries (HICs) show that NGS is cost-effective and reliable in diagnosing adult neurogenetic disorders (NGDs). African populations harbour vast genomic diversity, but there is limited knowledge on the molecular basis of NGDs affecting these populations due to lack of access to the necessary technology. The primary objective of this retrospective study was to describe the clinical utility of NGS panels in an African low-middle income country (LMIC). It included data of 74 adult participants seen at the multidisciplinary neurogenetic clinic at Tygerberg Hospital, South Africa, over a 4 – year period. Forty-three symptomatic index cases underwent NGS panel testing, while 31 relatives received targeted familial variant testing based on specific indications relevant to each case. Twenty-two different disease group-specific NGS panels were requested, spanning the NGD phenotypic spectrum. The diagnostic yield (DY) in index cases was 39.5% (17/43). Four relatives were clinically affected, and all tested positive for the familial-specific variant. This study demonstrated the DY achieved with NGS testing in an LMIC adult neurogenetic cohort, was comparable to DYs previously reported in HICs. These results argue for the use of NGS panels as first-tier testing in resource constrained LMICs, to limit lengthy diagnostic odysseys and unnecessary investigations. A definitive molecular diagnosis enables evidence-based management, surveillance, genetic counselling, and familial variant screening for relatives. Lastly, it assists with enrolment into clinical trials focussed on the development of precision medicine.

## Introduction

Neurogenetic disorders (NGDs) include a vast group of diseases with marked phenotypic and genetic heterogeneity which can present in the pediatric and adult setting [1–8]. Adult-onset NGDs can be clinically grouped into several diagnostic subcategories, including movement disorders, neuromuscular disorders, neuropathies, neurodegenerative disorders, mitochondrial disorders, metabolic disorders, neurotransmitter disorders, and epilepsies. Affected patients and their families often go through a lengthy diagnostic process as establishing a definitive diagnosis requires extensive clinical, radiological, and genetic investigations. Clinically, factors such as reduced penetrance and variable expressivity can make it challenging to identify a NGD based solely on family history [9].

Next generation sequencing (NGS) and large-scale genomic analyses have revolutionized diagnostics and led to advances in understanding the underlying genomic contribution to disease, particularly in the realm of NGDs [1, 3, 5, 8, 10]. These diagnostic tools have created opportunities to integrate genomic data into clinical practice, improve the diagnostic process and solve the problem of the lengthy diagnostic odysseys in rare NGDs [1, 5, 10, 11]. Precise and timely diagnosis of these disorders is essential to offer appropriate treatment and accurate genetic counseling.

The utility and diagnostic yield (DY) of NGS in monogenic NGDs have been described in numerous studies [1, 5, 10]. Global consensus suggests that targeted NGS gene panels can be used as a first-tier test for patients with a suspected complex NGD, and if results are negative, exome sequencing or genome sequencing can be considered as second tier testing platforms [5, 11]. In a study by Marques Matos et al. [12], ~33.2% of patients, including adults and children with neurological disorders of undetermined etiology, received a definitive diagnosis by using NGS panels [12].

African populations harbor the greatest human genomic diversity, but despite this, genomic studies, and current knowledge on NGDs affecting these populations are limited [3]. In low-middle income countries (LMICs) the availability of NGS is largely limited by factors such as cost and lack of infrastructure and expertise in the public health care system. The South African public health sector provides medical care to the majority of South Africans, and the limited availability of comprehensive genetic testing decreases the overall DY in adults affected by NGDs.

The multidisciplinary adult neurogenetic clinic at Tygerberg Hospital (TBH) in South Africa has had access to NGS panel diagnostic testing since 2019. Here, we present the results of an audit of this service over a 4-year period and highlight DYs and recommendations for incorporating NGS testing into other LMIC neurogenetic services.

## Materials and methods

### Study design and setting

This was a retrospective observational descriptive study, conducted in the adult neurogenetic clinic of TBH, Cape Town, South Africa. This academic hospital is the largest state hospital in the Western Cape and the second largest medical facility in the public healthcare sector in South Africa, providing medical care to a large and diverse low- to middle income population. Adult patients with suspected NGDs, are referred to the TBH adult neurology service from primary, secondary, and tertiary level care, as well as from the private sector. If an NGD is suspected based on family history, medical history, clinical signs, symptoms, and neuroimaging findings, the patient is evaluated in the neurogenetic clinic by a multidisciplinary team consisting of Neurologists, Medical Geneticists and Genetic Counselors. NGS panel testing is requested in patients where the differential diagnosis points to a NGD amenable to diagnosis by NGS panel. When applicable, a clinical assessment for at risk family members is arranged to determine whether they are affected and offer diagnostic, predictive, carrier or VUS resolution testing.

### Study sample

The study included all adults (18 years and older) who underwent NGS panel testing during the 4-year study period (from January 1, 2019, to December 31, 2022) through the adult neurogenetic clinic at TBH. During this period, the adult neurology outpatient service assessed an estimated total of 4800 new patients, of whom 240 patients with suspected NGDs (60 per year) were evaluated by a multidisciplinary team at the neurogenetic clinic.

### Data collection and analysis

Following pre-test genetic counseling and informed consent, genomic DNA was collected using buccal swabs, assisted saliva kits, or venous blood draws. NGS panel testing was performed by Invitae Corporation (San Francisco, California, USA), a CAP-accredited and CLIA-certified clinical diagnostic laboratory. The analysis included full-gene sequencing and deletion/duplication (CNV) analysis using NGS technology aligned to the GRCh37 reference genome. The assay has demonstrated >99% analytical sensitivity and specificity for single nucleotide variants, small insertions/deletions (<15 bp), and exon-level CNVs based on validation studies.

Participants were selected for testing following multidisciplinary evaluation, based on clinical suspicion of a NGD. Test results were categorized as: (i) positive-affected, when a pathogenic or likely pathogenic variant consistent with the phenotype was identified; (ii) positive-carrier, when a single pathogenic or likely pathogenic variant was found in a gene for an autosomal recessive condition without a second causative variant; (iii) inconclusive, when only a VUS was detected; and (iv) negative, when no clinically significant variants were identified.

Certain neurogenetic conditions that require alternative diagnostic methods such as repeat expansion disorders (e.g., Huntington’s disease, myotonic dystrophy, spinocerebellar ataxias) and *PMP22* duplications associated with Charcot-Marie-Tooth disease type 1 A, were evaluated separately through the South African National Health Laboratory Service (NHLS). These local diagnostic results were not included in the current study. Details of NHLS available tests and turnaround times are provided in Supplementary Table 1.

## Results

### Participant characteristics

This study included 74 participants: 43 clinically affected index cases who underwent NGS panel testing, and 31 family members who received targeted testing for known familial variants. Genetic testing was pursued based on a range of neurological indications, which were grouped into major clinical categories to guide panel selection. These categories included neuromuscular disorders (e.g., muscular dystrophies, hereditary neuropathies), movement disorders (e.g., hereditary ataxias, dystonias, early-onset parkinsonism), and adult-onset neurodegenerative conditions (e.g., leukodystrophies, hereditary spastic paraplegias, frontotemporal dementia). Testing was primarily performed using a range of Invitae diagnostic panels tailored to these phenotypes, such as the Comprehensive Neuropathies panel, Neuromuscular Disorders panel, Dystonia Comprehensive panel, and the Hereditary Amyotrophic Lateral Sclerosis, Frontotemporal Dementia, and Alzheimer Disease panel. A detailed list of panel types, associated disorder groups, and included genes is provided in Supplementary Table 2.

Table 1 presents the demographic characteristics of the study cohort. At the time of NGS panel analysis, the median age of participants was 36 years (interquartile range: 25–53 years), with ages ranging from 18 to 75 years. The cohort exhibited a female-to-male ratio of approximately 1.5:1. Self-reported ancestry indicated that the majority of participants identified as Caucasian (59.5%), followed by individuals of Mixed Ancestry South African (24.3%), African (9.5%), and Indian (6.7%) descent.

**Table 1:** Participant characteristics.

CATEGORY	DETAILS
Total Participants	74
COMBINED FEMALE AND MALE AGE STATISTICS	
Age Range	18 to 75 years
Median Age	36 years (IQR 25–53)
AGE DISTRIBUTION	
18–19 years	**6** (8.1%)
20–29 years	**23** (31.0%)
30–39 years	**14** (18.9%)
40–49 years	**5** (6.8%)
50–59 years	**19** (25.7%)
60 and above	**7** (9.5%)
ANCESTRY PROFILE	
African	**7** (9.5%)
Mixed Ancestry South African	**18** (24.3%)
Caucasian	**44** (59.5%)
Indian	**5** (6.7%)
DIAGNOSTIC YIELD IN INDEX PARTICIPANTS (*n* = 43)	
Positive Pathogenic Result	**17** (39.5%)
Positive Carrier Status	**7** (16.3%)
Variant of Uncertain Significance (VUS)	**12** (27.9%)
Negative Result	**7** (16.3%)
FAMILY VARIANT TESTING (*n* = 31)	
Familial Carrier Status Screening	**13** (41.9%)
Familial Symptomatic Diagnostic Test	**4** (12.9%)
Familial Asymptomatic Predictive Test	**11** (35.5%)
Familial VUS Resolution	**3** (9.7%)
FAMILY VARIANT TEST OUTCOMES	
Variant Detected (Carrier Screening)	Parent (4), Sibling (3)
Variant Not Detected (Carrier Screening)	Offspring (2), Parent (3), Sibling (1)
Variant Detected (Symptomatic Diagnostic Test)	Parent (1), Sibling (3)
Variant Detected (Asymptomatic Predictive Test)	Offspring (1), Sibling (1)
Variant Not Detected (Asymptomatic Predictive Test)	Offspring (4), Parent (2), Sibling (3)
Variant Detected (VUS Resolution)	Parent (2)
Variant Not Detected (VUS Resolution)	Offspring (1)

Bold numbers indicate actual number, with percentage of total in brackets.

### Diagnostic yield in index cases

The diagnostic yield of NGS panels in this cohort is summarized in Tables 1 and 2. The overall diagnostic yield across the 43 index cases was 39.5% (17/43). Among the 22 Neuromuscular disorder panels requested, 10 returned a definitive molecular diagnosis, yielding a diagnostic rate of 45.5%. Similarly, 4 out of 19 neuropathy panels resulted in a confirmed diagnosis, corresponding to a DY of 21%. No pathogenic or likely pathogenic variants were identified in panels requested for movement disorders, developmental brain abnormalities, or metabolic disorders. This is consistent with the lower pre-test probability for these conditions, as they are less commonly monogenic in origin compared to neuromuscular disorders. However, the interpretation of DYs in specific phenotypic subgroups is limited by the small sample sizes. Carrier status for pathogenic variants was detected in 7 of the 43 cases (16.3%), while VUSs were the only findings in 12 cases (27.9%). In the remaining 7 cases (16.3%), NGS panel analysis returned negative results.

**Table 2:** Phenotype and pathogenic variants in affected index cases.

NR	PHENOTYPIC FEATURES	GENE & VARIANT	OMIM	INHERITANCE/ ZYGOSITY	ACMG CLASSIFICATION and CRITERIA	PRECISION THERAPY	THERAPY IMPLEMENTED OR ADJUSTED
**1**	Muscle weakness of the face, neck flexors, and distal limbs. Subtle glove-and-stocking sensory changes, with normal nerve conduction studies. The family history suggested an autosomal dominant inheritance pattern.	*NEB* Deletion (Exons 14-77) Variation ID: 831138 SCV001195028.1	# 161650	Autosomal Dominant Heterozygous	Pathogenic	None	Treatment is predominantly rehabilitative and symptomatic, as there are currently no effective disease-modifying therapies to alter its natural progression.
**2**	The clinical presentation is consistent with a suspected diagnosis of retinal vasculopathy and cerebral leukoencephalopathy with systemic manifestations (RVCL-S).	*TREX1* NM_033629.6 Exon 2, c.795_802dup (p.Arg268Lysfs*12) Variation ID: 1463131 SCV002245235.1	# 192315	Autosomal Dominant Heterozygous	Likely Pathogenic PVS1 PM2	Currently, a pilot study for treating RVCL-S with aclarubicin has been started.	Treatment is predominantly rehabilitative and symptomatic, as there are currently no effective disease-modifying therapies to alter its natural progression.
**3**	Early onset and slowly progressive muscle weakness. No Neuroimaging.	*TTN* NM_001267550.2: c.63577dup (p.Arg21193fs) Variation ID: 1506927 SCV002287929.4	# 603689	Autosomal Dominant Heterozygous	Likely pathogenic PVS1 PM2 PP5	None	At present no disease-modifying therapy exists. Management is supportive.
**4**	Progressive proximal weakness in both the lower and upper limbs. EMG showed myopathic features. No Neuroimaging.	*GAA* NM_000152.5: c.-32-13T>G (Intronic) Variation ID: 4027 SCV000626604.10	# 232300	Autosomal Recessive Compound Heterozygous	Pathogenic PM3 PM2 BP7 PP5	Enzyme replacement therapy is currently the most reliable treatment	Enzyme replacement therapy started.
		*GAA* NM_000152.5: c.1441T>C (p.Trp481Arg) Variation ID: 189007 SCV000956690.7			Pathogenic PM3 PS3 PS1 PM1 PP2 PM2 PP3 PP5		
**5**	Delayed onset of walking, frequent falls and progressive limb weakness (distal > proximal). Nerve conduction studies: Sensorimotor polyneuropathy with mixed demyelinating and axonal features. No Neuroimaging.	*MORC2* NM_001303256.3: c.707A>G (p.Glu236Gly) Variation ID: 254251 SCV000655832.8	# 616688	Autosomal Dominant Heterozygous	Pathogenic PS4 PM2 PM5 PP2 PP5	None	Treatment is predominantly rehabilitative and symptomatic, as there are currently no effective disease-modifying therapies to alter its natural progression.
**6**	Lower limb weakness and spasticity, with a similarly affected sibling. No Neuroimaging.	*SPG11* NM_025137.4: c.2612dup (p.Ser871fs) Variation ID: 856716 SCV001227014.6	# 604360	Autosomal Recessive Compound Heterozygous	Pathogenic PM3 PVS1 PM2 PP5	None	At present, there is no specific treatment to prevent or reverse nerve degeneration in Hereditary Spastic Paraplegia.
		*SPG11* NM_025137.4: c.5989_5992del (p.Leu1997fs) Variation ID: 41335 SCV001394007.6			Pathogenic PM3 PP1 PVS1 PM2 PP5		
**7**	Progressive weakness and spasticity of the hands and lower limbs. No Neuroimaging.	*SPG11* NM_025137.4: c.2716del (p.Gln906fs) Variation ID: 41297 SCV002188001.4	# 604360	Autosomal Recessive Compound Heterozygous	Pathogenic PM3 PVS1 PM2 PP5	None	At present, there is no specific treatment to prevent or reverse nerve degeneration in Hereditary Spastic Paraplegia.
		*SPG11* NM_025137.4: c.3997A>T (p.Lys1333Ter) Variation ID: 1456680 SCV002243137.4			Pathogenic PVS1 PM2 PP5		
**8**	Progressive distal weakness in all limbs, dysarthria, nasal speech, and mild scapular winging. Normal ECG.	*MYOT* NM_006790.3: c.116C>T (p.Ser39Phe) Variation ID: 5839 SCV003439235.3	# 609200	Autosomal Dominant Heterozygous	Pathogenic PS4 PP1 PM2 PP5	None	Treatment is predominantly rehabilitative and symptomatic, as there are currently no effective disease-modifying therapies to alter its natural progression.
**9**	Congenital limb contractures, progressive motor decline and loss of ambulation in early childhood. Normal Neuroimaging.	*COL6A2* NM_001849.4: c.802-2A>G (Splice acceptor) Variation ID: 290251 SCV003232417.3	# 620725	Autosomal Dominant Heterozygous	Likely pathogenic PVS1 PM2 PP5	None	Treatment is predominantly rehabilitative and symptomatic, as there are currently no effective disease-modifying therapies to alter its natural progression.
**10**	Progressive muscle weakness with loss of ambulation. A similarly affected sibling. Creatine kinase levels significantly elevated. No Neuroimaging.	*DYSF* NM_001130987.2: c.5458-1G>A (Splice acceptor) Variation ID: 284470 SCV001574048.5	# 253601	Autosomal Recessive Homozygous	Pathogenic PM3 PVS1 PM2 PP5	None	There is no approved therapy for dysferlinopathy. Treatment is symptomatic only.
**11**	Cardiomyopathy and arrhythmia, with bilateral proximal weakness of the lower limbs and right scapular weakness. No Neuroimaging.	*DMD* Gain (Exons 2-7) Variation ID: 455821 SCV000625794.9	# 300376	X - Linked Hemizygous	Pathogenic HI SCORE:3 Sufficient Evidence for Haploinsufficiency	Dystrophin Restoration Therapies	Treatment of Cardiomyopathy and scoliosis. Corticosteroid therapy to treat weakness.
**12**	Progressive muscle weakness affecting the limb-girdle musculature of the lower limbs. No Neuroimaging.	*CAPN3* NM_000070.3: c.1117T>C (p.Trp373Arg) Variation ID: 217145 SCV000645464.6	# 253600	Autosomal Recessive Homozygous	Pathogenic PS4 PP1 PS3 PM2 PP2 PP3 PP5	None	Treatment is predominantly rehabilitative and symptomatic, as there are currently no effective disease-modifying therapies to alter its natural progression.
**13**	Distal weakness with an autosomal dominant inheritance pattern. No Neuroimaging.	*PMP22* Gain (Entire coding sequence) Variation ID: 218289 SCV000219138.4	# 118220	Autosomal Dominant Heterozygous	Pathogenic HI SCORE:3 Sufficient Evidence for Haploinsufficiency	None	Treatment is predominantly rehabilitative and symptomatic, as there are currently no effective disease-modifying therapies to alter its natural progression.
**14**	Myotonia involving limbs and facial muscles. EMG confirmed frequent myotonic discharges. No Neuroimaging.	*CLCN1* NM_000083.3: c.1437_1450del (p.Pro480fs) Variation ID: 279778 SCV000636291.10	# 255700	Autosomal Recessive Compound Heterozygous	Pathogenic PM3 PVS1 PM2 PP5	None	Myotonic stiffness may respond to sodium channel blockers or other pharmacologic treatment options.
		*CLCN1* NM_000083.3: c.1580T>G (p.Ile527Ser) Variation ID: 860935 SCV001232393.6			Pathogenic PM3 PP3 PM2 PM5 PP2 PP5		
**15**	Cerebral hemorrhaging and progressive cognitive decline. A similarly affected parent suggested an autosomal dominant inheritance. No Neuroimaging.	*APP* NM_000484.4: c.2077G>C (p.Glu693Gln) Variation ID: 18087 SCV001587274.5	# 605714	Autosomal Dominant Heterozygous	Pathogenic PS4 PP1 PS3 PM2 PM1 PM5 PP3 PP5	None	Management is often based on presenting symptoms.
**16**	Neuropathy, ataxia, and vision loss. A positive family history was noted. No Neuroimaging.	*AIPL1* NM_014336.5: c.834G>A (p.Trp278Ter) Variation ID: 5565 SCV000833757.7	# 604393	Autosomal Recessive Homozygous	Pathogenic PM3 PP1 PS3 PVS1 PM2 PP5	Successful gene supplementation therapy in experimental models of *AIPL1* - associated LCA.	Management is symptomatic.
**17**	Gait difficulties, with involvement of both proximal and distal muscle groups with dysphagia. Central core myopathy on muscle biopsy.	*MYH7* NM_000257.4: c.4850_4852del (p.Lys1617del) Variation ID: 190401 SCV000623724.7	# 160500	Autosomal Dominant Heterozygous	Pathogenic PS4 PP1 PM2 PM4 PM1 PP5	None	Treatment is predominantly rehabilitative and symptomatic, as there are currently no effective disease-modifying therapies to alter its natural progression.

### Pathogenic variants identified in index cases

Table 2 summarizes the clinical features and pathogenic variants identified in participants for whom a definitive molecular diagnosis was established. Of the 17 individuals with confirmed diagnoses, 52.9% (9/17) had conditions inherited in an autosomal dominant manner, while 41.2% (7/17) were affected by autosomal recessive disorders, comprising 3 cases with homozygous variants and 4 with compound heterozygous variants. One participant (5.9%) was diagnosed with an X-linked condition.

### Specific case examples

To illustrate the clinical and diagnostic value of genomic testing, several representative cases from the cohort are described in more detail below:

Participant 1 (Table 2) is a female of European ancestry, who presented with asymmetric scapular winging and muscle weakness affecting her face, neck flexors, and distal limbs. She exhibited subtle glove-stocking sensory changes, although her nerve conduction studies were normal. A novel heterozygous pathogenic deletion (Exons 14–77) in *NEB* was identified through the incorporation of CNV analysis tools into NGS variant-calling pipelines. Her family history strongly suggested an autosomal dominant inheritance pattern, as her mother, brother, and maternal grandmother were similarly affected. The same pathogenic variant was confirmed in her mother and brother. However, at the time of testing, only preliminary evidence supported the link between this variant and autosomal dominant nemaline myopathy, leading to her initial classification as a carrier for autosomal recessive *NEB*-related conditions. Since then, evidence has emerged showing that structural variants in *NEB* can cause an autosomal dominant condition, likely through a dominant-negative mechanism, with numerous families identified globally [13].This case underscores the evolving nature of variant classification, highlighting how variants can be reclassified as benign or pathogenic as more evidence becomes available. It also emphasizes the critical role of integrating CNV analysis tools into NGS pipelines, particularly in LMICs where alternate CNV techniques may not be available. This approach not only enhances DY without the need for additional technologies like chromosomal microarrays but also enables the identification of novel variants in underrepresented populations.

The clinical presentation of Participant 2 (Table 2) was consistent with suspected retinal vasculopathy and cerebral leukoencephalopathy with systemic manifestations (RVCL-S). The *TREX1*, Exon 2, c.795_802dup (p. Arg268Lysfs*12) variant was initially classified as a VUS. However, given that previously reported variants associated with this condition are also heterozygous frameshift variants and based on ACMG criteria (PSV1 and PM2), the variant is classified as likely pathogenic and is considered causative for her phenotype [14].

Participant 3 (Table 2) presented with early-onset, slowly progressive muscle weakness. The Invitae Limb-Girdle Muscular Dystrophy panel identified a heterozygous likely pathogenic variant in *TTN*. This sequence change creates a premature translational stop signal, and it is expected to create a truncated *TTN* protein. *TTN* is the largest gene in the human genome and is associated with a spectrum of autosomal dominant and recessive cardiac and neuromuscular conditions. However, this case is not considered solved by the detected *TTN* variant, as her phenotype does not align with tardive tibial muscular dystrophy, which is characterized solely by a distal lower limb phenotype. Autosomal dominant hereditary myopathy with early respiratory failure has only been associated with gain of function *TTN* pathogenic variants located in the 119th fibronectin-3 domain. This case underscores the critical importance of genotype-phenotype correlation. Even when a report identifies a pathogenic variant or suggests a diagnosis, the variant may not explain the clinical presentation completely, or in part and could be incidental. Additionally, these cases highlight the importance of involving a multidisciplinary team including neurologists, medical geneticists, and genetic counselors, in interpreting results to ensure accurate diagnoses and appropriate genetic counseling.

Importantly, molecular genetic diagnoses create opportunities for precision treatment.

A timely diagnosis of Pompe disease in Participant 4 (Table 2) was pivotal, enabling the initiation of enzyme replacement therapy, which has the potential to significantly modify the disease course and enhance the patient’s quality of life. Notably, this diagnosis represents the only case in this study where a disease-modifying treatment was explicitly implemented. However, a critical point for discussion is that precision treatments are on the horizon, albeit potentially distant in South Africa, and obtaining a precise genetic diagnosis positions clinicians to assemble trial-ready cohorts for future therapeutic advancements.

### Targeted family testing results

Among the 31 participants who underwent targeted familial variant NGS testing, 4 were clinically affected, all of whom received a confirmed molecular diagnosis through diagnostic testing. Predictive testing was carried out for 11 asymptomatic individuals, resulting in 2 confirmed diagnoses. Carrier status screening was conducted for 13 individuals, while testing to resolve VUSs was performed for 3 participants. Table 1 provides a summary of the indications for familial variant testing among these 31 relatives and their relationships to the index cases.

## Discussion

This study aimed to evaluate the DY of NGS gene panels in a South African adult patient cohort with suspected NGDs attending a tertiary neurogenetics service in an LMIC setting. The overall study DY was 39.5% (17/43 patients). This is comparable with the mean diagnostic yield of 25.6% detected in other studies conducted in high-income countries (HICs). Several studies have investigated the diagnostic yield of molecular testing in adult neurogenetic disorders. In a study by Marques Matos et al. [12], approximately 33.2% of patients, including adults and children with neurological disorders of undetermined aetiology, received a definitive diagnosis by using NGS panels [12]. Winder et al. [15], conducted a study to evaluate the clinical utility of NGS multigene analysis in over 25,000 patients (<1 – 96 years) with neuromuscular disorders. By using NGS-based gene panels, a definitive molecular diagnosis was obtained for 5055 of the 25,356 individuals. This represents an overall diagnostic yield of 20%, and a range of 4–33%, depending on the panel used in individuals with neuromuscular diseases [15]. Kwan et al. [16] did a mini-review study to describe the use of gene panels in the diagnosis of neuromuscular disorders in children and adults. They found that in the studies using comprehensive gene panels for undifferentiated categories of neuromuscular disorders, the yield has ranged from 12.9% to 48.8% [16]. A systematic review done by Gorcenco et al. 2020, identified 28 studies that evaluated the efficacy of NGS in diagnosing movement disorders in children and adults [17, 18].

Limited studies in other LMICs are available for NGDs in adult cohorts. Ganapathy et al. [19] reported an overall diagnostic yield of 40%, however most of the cohort were pediatric patients. Data from their study strongly suggested that the NGS-based multi-gene tests should be considered as the first-tier genetic test for neurological disorders in India [19] (Table 3).

**Table 3:** Diagnostic yield for NGS panels in neurogenetic disorders in other countries.

AUTHOR	COUNTRY	CONDITION INVESTIGATED	AGE GROUP	DIAGNOSTIC YIELD
Marques Matos et al. [12]	Portugal	Neurological disorders	Children & adults	33.2%
Winder et al. [15]	USA	Neuromuscular disorders	Children & adults	4%–33%
Kwan et al. [16]	Singapore	Neuromuscular disorders	Children & adults	12.9% to 48.8%
Kwan et al. [16]	Singapore	Muscle disorders	Children & adults	13% to 79%
Kwan et al. [16]	Singapore	Inherited neuropathies	Children & adults	6%–46%
Kwan et al. [16]	Singapore	Hereditary spastic paraplegias	Children & adults	20%
Gorcenco et al. [18]	Sweden	Familial Parkinson disease	Adults	10.1%
Gorcenco et al. [18]	Sweden	Early-onset Parkinson disease	Children & adults	15.7%
Gorcenco et al. [18]	Sweden	Dystonia	Children & adults	11.7% –37.5%
Gorcenco et al. [18]	Sweden	Ataxia/spastic paraplegia	Children & adults	12.1–61.8%
Gorcenco et al. [18]	Sweden	Combined movement disorders	Children & adults	11.3% –28%
Ganapathy et al. [19]	India	Neurological disorders	Children & adults	40%

In this study, 16.3% (7/43) of index cases were found to carry heterozygous pathogenic variants in genes associated with autosomal recessive conditions. These findings are most consistent with carrier status, rather than a definitive molecular diagnosis. However, such results may still hold clinical relevance, particularly when the individual’s phenotype partially aligns with the associated condition. In these cases, a second pathogenic variant may have been missed due to test limitations (e.g., deep intronic or regulatory variants not covered by the panel), or may have been detected but classified as a VUS.

Participant 18 (Supplementary Table 3) exemplifies this scenario, where a single VUS may still carry potential diagnostic importance depending on future reclassification or emerging evidence. She presented with ataxia, spasticity, and cognitive decline beginning at age 23, along with diffuse cerebral white matter loss on MRI, features suggestive of a leukodystrophy. Molecular testing identified two variants in the *EIF2B5* gene: one likely pathogenic splice site variant (c.1654+1G>T (splice donor) and one missense VUS (c.383A>G (p.Tyr128Cys)). While a definitive molecular diagnosis could not be made, the clinical and genetic findings are suggestive of *EIF2B5*-related vanishing white matter disease. This case highlights how VUS findings, particularly when occurring alongside a known pathogenic variant, may hold evolving diagnostic significance, warranting close follow-up and potential reclassification over time.

In this study, a total of 67.4% (29/43) index cases had at least one VUS detected. In 27.9% (12/43) of index cases, only VUSs were reported, and a definitive diagnosis could not be achieved. This may be explained by our population being underrepresented in global databases and emphasizes the importance of further studies, and creation of local genomic databases. Participant 19 (Supplementary Table 3) is an African male who presented with slowly progressive spastic paraparesis from the age of 13 years, and he was homozygous for the c.1507A>G (p. Lys503Glu) variant in *CYP7B1*, which was classified as a VUS. His phenotype was compatible with autosomal recessive hereditary spastic paraplegia type 5 A, associated with bi-allelic variants in the *CYP7B1* gene. His asymptomatic mother was found to be a heterozygous carrier for the same variant, but his father was not available for testing. Family testing to try and resolve variants of uncertain significance was limited due to unavailability of parents/other appropriate family members.

## Conclusion

To the best of our knowledge, this is the first study that specifically investigated the DY of commercially available NGS panels in a cohort attending a tertiary adult neurogenetic service in an LMIC. The high overall DY in this study is comparable to previously reported adult neurogenetic cohorts in HICs. This study illustrates that the use of NGS panel testing can be successfully implemented in the clinical practice of LMICs and assist in the diagnostic workup in patients affected by complex NGDs. The high detection rate of VUSs highlights that our population is underrepresented in global databases, and further studies are needed to resolve these variants. A definitive diagnosis is the cornerstone of clinical management, and we are hopeful that NGS panel testing will be incorporated as a useful diagnostic modality in neurology practice in South Africa.

## Supplementary information

Supplementary Table 1

Supplementary Table 2

Supplementary Table 3

## Data Availability

All data generated are available in this article and supplementary files. Testing was conducted at Invitae (USA), and variants have been submitted to ClinVar as per Invitae practice. ClinVar submission accession numbers are included in Table 2 and Supplementary table 3.
