# Supplementary material for: The utility of next generation sequencing targeted multigene panels in the Adult Neurogenetic Clinic at Tygerberg Hospital, South Africa
Source: Eur J Hum Genet. 2025 Jun 25;33(9):1144–52. doi: 10.1038/s41431-025-01900-2 (PMC12402217; doi:10.1038/s41431-025-01900-2)
Supplement: Supplementary file 1 — Supplementary Table 1 [file 41431_2025_1900_MOESM1_ESM.pdf]

SUPPLEMENTARY TABLE 1<br>LOCALLY AVAILABLE GENETIC TESTS FOR ADULT-ONSET NEUROGENETIC DISORDERS			
NEUROGENETIC DISORDER	TEST	TAT (WEEKS)	NHLS
**Charcot-Marie-Tooth Type 1A**	*PMP-22* gene (1.5Mb duplication) MLPA	8 to 16	WITS
***FKRP*-related muscular dystrophy**	*FKRP* gene variants	8 to 16	WITS
**Hereditary Neuropathy with Liability to Pressure Palsies**	*PMP-22* gene (1.5Mb deletion) MLPA	8 to 16	WITS
**Dystrophinopathy**	*DMD* exonic deletions/duplications (MLPA)	8 to 24	WITS<br>UCT
**Myotonic dystrophy Type 1**	*DMPK* (triplet expansion) PCR	6 to 12	WITS
**Kennedy's Disease**	*AR* gene (triplet repeat expansion) PCR	6	WITS
**Huntington disease**<br>**Huntington-like disease type 2**	*HTT* and *JPH3*<br>(triplet repeat expansion) PCR	6 to 12	WITS<br>UCT
**Spinocerebellar ataxias** (SCA)	*SCA* 1, 2, 3, 6, 7 and 17<br>(triplet repeat expansions) PCR	6	UCT
**Dentatorubral-pallidoluysian atrophy** (DRPLA)	*ATN1* (triplet repeat expansion) PCR	6	UCT
**Friedreich ataxia** (FRDA)	*FXN* (triplet repeat expansion) PCR	6	UCT
**Spinal Muscular Atrophy** (SMA)	*SMN1* (Exon 7 deletion)	4	UCT
**Cerebral arteriopathy with subcortical infarcts and leukoencephalopathy 1** (CADISIL)	*NOTCH3*	6	UCT
**MELAS**<br>(Mitochondrial Encephalomyopathy with Lactic Acidosis and Stroke-like Episodes)	m.3243A>G variant only or in addition to *MTTL1* sequencing	6	UCT
**Mitochondrial DNA full sequencing**<br>**Nuclear genes for mtDNA maintenance disorders**<br>(MELAS, MERRF, Leigh (MILS), LHON, NARP, MIDD, KSS, CPEO, CIPO, DEAF, SNHL, KS, etc.)	Full mtDNA sequencing	3-8 months	UCT

**TAT**	Turnaround time
**WITS**	University of the Witwatersrand
**UCT**	University of Cape Town
**MLPA**	Multiplex-ligation dependent probe amplification
**PCR**	Polymerase chain reaction
