# Supplementary material for: The utility of next generation sequencing targeted multigene panels in the Adult Neurogenetic Clinic at Tygerberg Hospital, South Africa
Source: Eur J Hum Genet. 2025 Jun 25;33(9):1144–52. doi: 10.1038/s41431-025-01900-2 (PMC12402217; doi:10.1038/s41431-025-01900-2)
Supplement: Supplementary file 2 — Supplementary Table 2 [file 41431_2025_1900_MOESM2_ESM.pdf]

SUPPLEMENTARY TABLE 2<br>DIFFERENT DISORDER GROUPS, PANELS PER GROUP AND GENES PER PANEL			
NUMBER	PANEL	GENES TESTED IN EACH PANEL	NUMBER OF GENES
**NEUROPATHIES AND RELATED DISORDERS**			
**1.**	**Invitae Comprehensive Neuropathies Panel**	*AARS, AIFM1, APOA1, ASAH1, ATL1, ATL3, ATP1A1, ATP7A, BAG3, BICD2, BSCL2, CHCHD10, COX6A1, CYP27A1, CYP7B1, DCTN1, DHTKD1, DNAJB2, DNM2, DNMT1, DRP2, DST, DYNC1H1, EGR2, ELP1, EXOSC9, FBLN5, FBXO38, FGD4, FIG4, GAN, GARS, GDAP1, GJB1, GLA, GNB4, GSN, HARS, HEXA, HINT1, HMBS, HSPB1, HSPB8, IGHMBP2, INF2, KIF1A, KIF5A, LITAF, LMNA, LRSAM1, MARS, MCM3AP, MED25, MFN2, MME, MORC2, MPZ, MTMR2, NDRG1, NEFH, NEFL, NGF, NTRK1, PDK3, PLEKHG5, PMP2, PMP22, POLG, POLG2, PRDM12, PRPS1, PRX, RAB7A, REEP1, RETREG1, SBF1, SBF2, SCN11A, SCN9A, SEPT9, SH3TC2, SIGMAR1, SLC12A6, SLC25A46, SLC52A2, SLC52A3, SLC5A7, SMN1, SMN2, SPG11, SPTLC1, SPTLC2, SURF1, TFG, TRIM2, TRPV4, TTR, UBA1, VAPB, VRK1, WNK1, YARS*	102
**2.**	**Invitae Hereditary Spastic Paraplegia Comprehensive Panel**	*ABCD1, ALDH18A1, ALS2, AP4B1, AP4E1, AP4M1, AP4S1, AP5Z1, ARG1, ARL6IP1, ATL1, ATP13A2, B4GALNT1, BSCL2, C12orf65, CAPN1, CPT1C, CYP27A1, CYP2U1, CYP7B1, DDHD1, DDHD2, ENTPD1, ERLIN1, ERLIN2, FA2H, FARS2, GBA2, GJC2, HACE1, HEXA, HSPD1, KCNA2, KDM5C, KIDINS220, KIF1A, KIF1C, KIF5A, L1CAM, MAG, NIPA1, NKX6-2, NT5C2, PLP1, PNPLA6, RAB3GAP2, REEP1, REEP2, RTN2, SACS, SLC16A2, SPART, SPAST, SPG11, SPG21, SPG7, TECPR2, TFG, UCHL1, VAMP1, WASHC5, ZFYVE26*	62
**3.**	**Invitae Charcot-Marie-Tooth Disease Comprehensive Panel**	*AARS, AIFM1, ATP1A1, BAG3, BSCL2, COX6A1, DHTKD1, DNAJB2, DNM2, DRP2, DYNC1H1, EGR2, FBLN5, FGD4, FIG4, GARS, GDAP1, GJB1, GNB4, HARS, HINT1, HSPB1, HSPB8, IGHMBP2, INF2, KIF5A, LITAF, LMNA, LRSAM1, MARS, MCM3AP, MED25, MFN2, MME, MORC2, MPZ, MTMR2, NDRG1, NEFH, NEFL, PDK3, PLEKHG5, PMP2, PMP22, PRPS1, PRX, RAB7A, SBF1, SBF2, SH3TC2, SLC25A46, SPG11, SURF1, TFG, TRIM2, TRPV4, YARS*	57
**4.**	**Invitae Hereditary Motor Neuropathy Panel**	*ASAH1, ATP7A, BICD2, BSCL2, CHCHD10, DCTN1, DNAJB2, DYNC1H1, EXOSC9, FBXO38, GARS, HEXA, HINT1, HSPB1, HSPB8, IGHMBP2, MORC2, PLEKHG5, REEP1, SIGMAR1, SLC5A7, SMN1, SMN2, TRPV4, UBA1, VAPB, VRK1*	27
**5.**	**Invitae Spinal Muscular Atrophy Panel**	*SMN1, SMN2*	2
**MOVEMENT DISORDERS**			
**6.**	**Invitae Dystonia Comprehensive Panel**	*ACTB, ADCY5, ANO3, ATP1A3, ATP7B, BCAP31, CIZ1, COL6A3, CYP27A1, GCH1, GNAL, GNAO1, HEXA, HPCA, KCNMA1, KCTD17, KMT2B, MECR, PANK2, PLA2G6, PNKD, PRKN, PRKRA, PRRT2, SGCE, SLC2A1, SLC30A10, SLC39A14, SLC6A3, SPR, TH, THAP1, TOR1A, TUBB4A, VAC14, VPS13A, VPS13D, XPR1*	38
**NEURODEGENERATIVE DISORDERS**			
**7.**	**Invitae Hereditary Amyotrophic Lateral Sclerosis, Frontotemporal Dementia and Alzheimer Disease Panel**	*ALS2, ANG, ANXA11, APP, CHCHD10, CHMP2B, DCTN1, ERBB4, FUS, GRN, HEXA, HNRNPA2B1, ITM2B, KIF5A, MAPT, OPTN, PFN1, PRNP, PSEN1, PSEN2, SETX, SNCA, SOD1, SORL1, SPG11, SQSTM1, TARDBP, TBK1, TFG, TREM2, UBQLN2, VAPB, VCP*	33

**8.**	**Invitae Hereditary Alzheimer's Disease Panel**	*APP, PSEN1, PSEN2*	3
		**DEVELOPMENTAL BRAIN ABNORMALITIES**	
**9.**	**Invitae Leukodystrophy and Genetic Leukoencephalopathy Panel**	*AARS, AARS2, ABAT, ABCA1, ABCD1, ABCD4, ABHD5, ACADS, ACBD5, ACER3, ACO2, ACOX1, ACP5, ACSF3, ACTA2, ACTB, ACY1, ADAR, ADGRG1, ADK, ADNP, ADSL, AGA, AHDC1, AHI1, AIFM1, AIMP1, AIMP2, ALDH18A1, ALDH3A2, ALDH5A1, ALDH6A1, ALG12, ALG13, ALG2, ALG6, ALG9, AMACR, AMPD2, AMT, ANK3, AP1S2, AP3B2, AP4B1, AP4E1, AP4M1, AP4S1, AP5Z1, APC2, APOPT1, ARCN1, ARFGEF2, ARHGAP31, ARHGEF9, ARNT2, ARSA, ARX, ASL, ASNS, ASPA, ASS1, ASXL1, ASXL2, ATP13A2, ATP5A1, ATP6AP2, ATP6V1A, ATP7A, ATP7B, ATP8A2, ATPAF2, ATRN, ATRX, AUH, B3GALNT2, B4GALNT1, BCAP31, BCAT2, BCKDHA, BCKDHB, BCL11B, BCS1L, BICD2, BMP4, BOLA3, BPTF, BRAT1, BTD, C12ORF57, C12ORF65, C19ORF12, C2CD3, CACNA1A, CACNA1E, CARS2, CBS, CC2D2A, CCDC88A, CDC42, CDKL5, CEP290, CHMP1A, CLCN2, CLCN4, CLCN7, CLN2 (TPP1), CLN3, CLN5, CLN6, CLN8, CLP1, CLPP, CNNM2, CNOT1, CNTNAP1, CNTNAP2, COA7, COASY, COG7, COL18A1, COL3A1, COL4A1, COL4A2, COLGALT1, COQ2, COQ6, COQ7, COQ8A, COQ9, COX10, COX14, COX15, COX20, COX6B1, COX7B, COX8A, CPLANE1, CPLX1, CPS1, CRAT, CREBBP, CRIPT, CRLF1, CSF1R, CSPP1, CTBP1, CTC1, CTDP1, CTNS, CTSA, CTSD, CUL4B, CYB5R3, CYFIP2, CYP27A1, CYP2U1, CYP7B1, D2HGDH, DAG1, DARS, DARS2, DBT, DCAF17, DCX, DDC, DDHD1, DDHD2, DDOST, DDX3X, DEAF1, DEGS1, DGUOK, DHCR24, DHFR, DHX37, DLD, DLL4, DMXL2, DNM1L, DNM2, DOCK6, DOCK7, DOLK, DONSON, DPAGT1, DPM1, DPM2, DPM3, DPYS, DYRK1A, EARS2, EDNRB, EHMT1, EIF2AK1, EIF2AK2, EIF2B1, EIF2B2, EIF2B3, EIF2B4, EIF2B5, ELOVL1, ELOVL4, ENTPD1, EOGT, EPG5, EPRS, ERCC1, ERCC2, ERCC3, ERCC6, ERCC8, ETFA, ETFB, ETFDH, ETHE1, EXOSC2, EXOSC3, EXOSC8, EXOSC9, FA2H, FAM126A, FAR1, FARS2, FARSB, FASTKD2, FBXL4, FDX2, FGFRL1, FH, FIG4, FKRP, FKTN, FLVCR2, FOLR1, FOXC1, FOXG1, FOXRED1, FUCA1, FUK, GAA, GABBR2, GABRA1, GABRB1, GABRB2, GABRB3, GALC, GALT, GAN, GATAD2B, GCDH, GDAP1, GFAP, GFM1, GFM2, GJA1, GJB1, GJC2, GLA, GLB1, GLDC, GLRX5, GLUL, GLYCTK, GM2A, GMNN, GNAO1, GNS, GOT2, GPHN, GRIN1, GRM7, GTF2H5, GTPBP2, HACE1, HCN1, HEPACAM, HERC1, HEXA, HEXB, HIBCH, HIKESHI, HK1, HLCS, HMGCL, HNRNPU, HSD17B10, HSD17B4, HSPD1, IARS, IARS2, IBA57, IDH2, IDS, IDUA, IER3IP1, IFIH1, INPP5E, IREB2, ISCA1, ISCA2, ITPA, ITPR1, IVD, JAM3, KARS, KAT6B, KATNB1, KCNJ10, KCNJ2, KCNMA1, KCNT1, KCTD7, KDM1A, KIAA0556, KIAA0586, KIAA0753, KIDINS220, KIF1A, KIF1C, KLHL7, KMT2E, L2HGDH, LAGE3, LAMA1, LAMA2, LAMB1, LARGE1, LARS2, LETM1, LIAS, LIPT1, LIPT2, LONP1, LRPPRC, LYRM7, MAG, MAGEL2, MAN2B1, MANBA, MARS2, MAST1, MAT1A, MCCC1, MCCC2, MCOLN1, MDH2, MECP2, MED17, MED25, MEF2C, MFSD2A, MFSD8, MGP, MICU1, MKS1, MLC1, MLYCD, MMACHC, MMADHC, MOCS1, MOCS2, MOGS, MORC2, MPLKIP, MPV17, MPZ, MRPL12, MRPL44, MRPS16, MRPS22, MRPS34, MSTO1, MTFMT, MTHFR, MTHFS, MTO1, MTOR, MTR, MTRR, MUT, NACC1, NADK2, NAGA, NAGLU, NAGS, NANS, NARS2, NAXD, NAXE, NDRG1, NDUFA1, NDUFA10, NDUFA11, NDUFA12, NDUFA13, NDUFA2, NDUFA4, NDUFA6, NDUFA9, NDUFAF1, NDUFAF2, NDUFAF3, NDUFAF4, NDUFAF5, NDUFAF6, NDUFB10, NDUFB11, NDUFB3, NDUFB8, NDUFB9, NDUFS1, NDUFS2, NDUFS3, NDUFS4, NDUFS6, NDUFS7, NDUFS8, NDUFV1, NDUFV2, NEK1, NFE2L2, NFU1, NGLY1, NKX6-2, NOTCH1, NPC1, NPC2, NPHP1, NRXN1, NSUN3, NT5C2, NTRK2, NUBPL, NUP62, OAT, OCRL, OPA1, OPA3, OSGEP, OTC, PACS1, PAFAH1B1, PAH, PANK2, PARS2, PAX1, PC, PCCA, PCCB, PCDH12, PCGF2, PDGFRB, PDHA1, PDHB, PDHX, PDP1, PET100, PEX1, PEX10, PEX11B, PEX12, PEX13, PEX14, PEX16, PEX19, PEX2, PEX26, PEX3, PEX5, PEX6, PEX7, PGAP1, PGK1, PHGDH, PHYH, PIGA, PIGB, PIGG, PIGL, PIGM,*	697

		PIGN, PIGP, PIGQ, PIGT, PIGU, PIGV, PIK3C2A, PIK3R2, PLA2G6, PLAA, PLEKHG2, PLP1, PMM2, PMP22, PNKP, PNPT1, POLG, POLG2, POLR1A, POLR1C, POLR3A, POLR3B, POMGNT1, POMK, POMT1, POMT2, PPP1R15B, PPP2R1A, PPP3CA, PPT1, PRF1, PRKDC, PRODH, PROSC, PRPS1, PRUNE1, PSAP, PSAT1, PSPH, PTEN, PTPN23, PURA, PUS3, PYCR2, QARS, QRSL1, RAB11B, RAB3GAP1, RAB3GAP2, RARS, RARS2, RBPJ, REPS1, RERE, RHOBTB2, RIN2, RMND1, RNASEH2A, RNASEH2B, RNASEH2C, RNASET2, RPGRIP1L, RPIA, RPS6KC1, RRM2B, RTTN, RXYLT1, SAMD9L, SAMHD1, SCN3A, SCO1, SCO2, SDHA, SDHAF1, SDHB, SDHD, SEPSECS, SERAC1, SETBP1, SGSH, SH3TC2, SHOC2, SHPK, SLC12A2, SLC12A5, SLC12A6, SLC13A3, SLC13A5, SLC16A2, SLC17A5, SLC19A3, SLC1A2, SLC1A3, SLC1A4, SLC25A1, SLC25A12, SLC25A15, SLC25A19, SLC25A22, SLC25A4, SLC25A42, SLC25A46, SLC2A1, SLC30A10, SLC33A1, SLC35A2, SLC39A14, SLC39A8, SLC46A1, SLC6A19, SLC6A8, SLC6A9, SLC9A1, SLC9A6, SMC1A, SNAP29, SNIP1, SNORD118, SNRPB, SNX14, SOD1, SON, SOX10, SOX2, SPART, SPAST, SPATA5, SPG11, SPG7, SPTAN1, SQSTM1, SRD5A3, SSR4, ST3GAL5, STAG2, STAMBP, STAT1, STAT2, STN1, STRADA, STX11, STXBP1, STXBP2, SUCLA2, SUCLG1, SUMF1, SUOX, SURF1, SYNE1, SYNJ1, TACO1, TAF2, TANGO2, TARS2, TBC1D24, TBCD, TBCE, TBCK, TBX1, TCF4, TCTN2, TIMM50, TIMMDC1, TM4SF20, TMEM106B, TMEM126B, TMEM165, TMEM216, TMEM67, TMEM70, TMTC3, TOE1, TP53RK, TPI1, TPK1, TRAPPC11, TRAPPC9, TREX1, TRMT10A, TRMT5, TSC1, TSC2, TSEN54, TTC19, TUBA1A, TUBA8, TUBB2A, TUBB2B, TUBB3, TUBB4A, TUBG1, TUFM, TWNK, TXN2, TYMP, UBA5, UBE2A, UBE3A, UFM1, UNC13D, UPB1, USP7, VARS2, VPS11, VPS13D, VPS33A, WARS2, WDR45, WDR73, WHSC1, WWOX, YARS, YME1L1, ZEB2, ZFYVE26, ZIC1, ZNF335	
**NEUROMUSCULAR DISORDERS**			
**10.**	**Invitae Comprehensive Neuromuscular Disorders Panel**	*ABHD5, ACAD9, ACADM, ACADVL, ACTA1, ADSSL1, AGK, AGL, AGRN, AHCY, ALDOA, ALG14, ALG2, AMACR, AMPD1, ANO5, ATP2A1, ATP7B, B3GALNT2, B4GAT1, BAG3, BIN1, C1QBP, CACNA1S, CAPN3, CASQ1, CAV3, CCDC78, CFL2, CHAT, CHKB, CHRNA1, CHRNB1, CHRND, CHRNE, CLCN1, CNTN1, COL12A1, COL13A1, COL6A1, COL6A2, COL6A3, COLQ, COQ2, COQ4, COQ7, COQ8A, COQ9, COX15, COX20, COX6B1, CPT1A, CPT2, CRYAB, CTDP1, DAG1, DDC, DES, DGUOK, DMD, DNA2, DNAJB6, DNM2, DOK7, DPAGT1, DPM1, DPM2, DPM3, DYSF, EMD, ENO3, ETFA, ETFB, ETFDH, FBXL4, FDX2, FHL1, FKBP14, FKRP, FKTN, FLAD1, FLNC, GAA, GATM, GBE1, GFER, GFPT1, GMPPB, GNE, GOSR2, GYG1, GYS1, HACD1, HADH, HADHA, HADHB, HMBS, HNRNPA2B1, HNRNPDL, ISCU, ISPD, ITGA7, KBTBD13, KCNJ2, KLHL40, KLHL41, LAMA2, LAMP2, LARGE1, LDB3, LDHA, LMNA, LMOD3, LPIN1, MAN2B1, MAP3K20, MATR3, MEGF10, MGME1, MICU1, MPV17, MTM1, MUSK, MYH2, MYH3, MYH7, MYL2, MYO18B,MYOT, MYPN, NEB, OPA1, OPA3, ORAI1, PDSS1, PDSS2, PFKM, PGAM2, PGK1, PGM1, PHKA1, PHKB, PLEC, PNPLA2, PNPLA8, POGLUT1, POLG, POLG2, POMGNT1, POMGNT2, POMK, POMT1, POMT2, PREPL, PUS1, PYGM, PYROXD1, RAPSN, RBCK1, RNASEH1, RRM2B, RXYLT1, RYR1, SCN4A, SDHA, SELENON, SGCA, SGCB, SGCD, SGCG, SIL1, SLC16A1, SLC18A3, SLC22A5, SLC25A20, SLC25A3, SLC25A4, SLC25A42, SLC5A7, SMCHD1, SMN1, SMN2, SMPX, SPEG, SQSTM1, STAC3, STIM1, SUCLA2, SUCLG1, SYT2, TANGO2, TAZ, TCAP, TIA1, TK2, TNNT1, TNPO3, TOR1AIP1, TPM2, TPM3, TRAPPC11, TRIM32, TRMT5, TSFM, TTN, TWNK, TYMP, VAMP1, VCP, VMA21, YARS2*	211
**11.**	**Invitae Comprehensive Muscular Dystrophy Panel**	*ANO5, B3GALNT2, B4GAT1, CAPN3, CAV3, CHKB, COL12A1, COL6A1, COL6A2, COL6A3, DAG1, DES, DMD, DNAJB6, DPM1, DPM2, DPM3, DYSF, EMD, FHL1, FKRP, FKTN, GAA, GMPPB, GOSR2, HNRNPDL, ISPD, ITGA7, LAMA2, LARGE1, LMNA, MYOT, PLEC, PNPLA2, POGLUT1, POMGNT1, POMGNT2, POMK, POMT1, POMT2, RXYLT1, SGCA, SGCB, SGCD, SGCG, SMCHD1, TCAP, TK2, TNPO3, TOR1AIP1, TRAPPC11, TRIM32, TTN*	53

**12.**	**Invitae Limb-Girdle Muscular Dystrophy Panel**	*ANO5, CAPN3, CAV3, DAG1, DES, DMD, DNAJB6, DYSF, FKRP, FKTN, GAA, GMPPB, GOSR2, HNRNPDL, ISPD, LAMA2, LMNA, MYOT, PLEC, PNPLA2, POGLUT1, POMGNT1, POMGNT2, POMK, POMT1, POMT2, SGCA, SGCB, SGCD, SGCG, SMCHD1, TCAP, TK2, TNPO3, TOR1AIP1, TRAPPC11, TRIM32, TTN*	38
**13.**	**Invitae Comprehensive myopathy panel**	*ACTA1, ADSSL1, AMPD1, ANO5, ATP2A1, BAG3, BIN1, CACNA1S, CASQ1, CAV3, CCDC78, CFL2, CLCN1, CNTN1, COL12A1, COL6A1, COL6A2, COL6A3, CPT2, CRYAB, DES, DNAJB6, DNM2, DYSF, FHL1, FKBP14, FLNC, GNE, GYG1, GYS1, HACD1, HNRNPA2B1, ISCU, KBTBD13, KCNJ2, KLHL40, KLHL41, LAMP2, LDB3, LMNA, LMOD3, MAP3K20, MATR3, MEGF10, MICU1, MTM1, MYH2, MYH7, MYL2, MYO18B, MYOT, MYPN, NEB, ORAI1, PYROXD1, RYR1, SCN4A, SELENON, SMPX, SPEG, SQSTM1, STAC3, STIM1, TAZ, TIA1, TK2, TNNT1, TPM2, TPM3, TTN, VCP, VMA21*	72
**14.**	**Invitae Distal Myopathy Panel**	*ANO5, BAG3, CAV3, CRYAB, DES, DNAJB6, DYSF, FHL1, FLNC, GNE, LDB3, MATR3, MYH7, MYOT, SQSTM1, TIA1, TTN, VCP*	18
**15.**	**Invitae Congenital Myopathy Panel**	*ACTA1, BIN1, CACNA1S, CCDC78, CFL2, CNTN1, COL12A1, COL6A1, COL6A2, COL6A3, DNM2, FKBP14, HACD1, KBTBD13, KLHL40, KLHL41, LMOD3, MAP3K20, MEGF10, MICU1, MTM1, MYH7, MYL2, MYO18B, MYPN, NEB, PYROXD1, RYR1, SELENON, SPEG, STAC3, TK2, TNNT1, TPM2, TPM3, TTN*	36
**16.**	**Invitae Hereditary Rhabdomyolysis Panel**	*ABHD5, ACAD9, ACADM, ACADVL, AGK, AGL, AHCY, ALDOA, AMACR, AMPD1, ANO5, ATP2A1, ATP7B, B3GALNT2, B4GAT1, C1QBP, CACNA1S, CAPN3, CASQ1, CAV3, CHAT, CHKB, COQ2, COQ4, COQ7, COQ8A, COQ9, COX15, COX20, COX6B1, CPT1A, CPT2, CTDP1, DAG1, DGUOK, DMD, DNA2, DNAJB6, DPM1, DPM2, DPM3, DYSF, EMD, ENO3, ETFA, ETFB, ETFDH, FBXL4, FDX2, FHL1, FKRP, FKTN, FLAD1, GAA, GATM, GBE1, GFER, GMPPB, GYG1, GYS1, HADH, HADHA, HADHB, HMBS, ISCU, ISPD, ITGA7, LAMA2, LAMP2, LARGE1, LDHA, LPIN1, MAN2B1, MGME1, MICU1, MPV17, MYH3, OPA1, OPA3, PDSS1, PDSS2, PFKM, PGAM2, PGK1, PGM1, PHKA1, PHKB, PNPLA2, PNPLA8, POLG, POLG2, POMGNT1, POMGNT2, POMK, POMT1, POMT2, PUS1, PYGM, RBCK1, RNASEH1, RRM2B, RXYLT1, RYR1, SCN4A, SDHA, SGCA, SGCB, SGCD, SGCG, SIL1, SLC16A1, SLC22A5, SLC25A20, SLC25A3, SLC25A4, SLC25A42, STAC3, SUCLA2, SUCLG1, TANGO2, TCAP, TK2, TNPO3, TRIM32, TRMT5, TSFM, TWNK, TYMP, YARS2*	129
**17.**	**Invitae Myotonia and Paramyotonia Congenita Panel**	*CLCN1, SCN4A*	2
**METABOLIC DISORDERS**			
**18.**	**Invitae Neurotransmitter Disorders Panel**	*ABAT, ALDH5A1, ALDH7A1, AMT, ARHGEF9, ASNS, ATAD1, DBH, DDC, DHFR, DNAJC12, GABBR2, GABRA1, GABRA2, GABRB1, GABRB3, GABRG2, GAD1, GCH1, GLDC, GLRA1, GLRB, GOT2, GPHN, GRIN2B, GRIN2D, MAOA, PCBD1, PHGDH, PNPO, PROSC, PSAT1, PSPH, PTS, QDPR, SLC18A2, SLC1A2, SLC1A4, SLC25A22, SLC6A1, SLC6A3, SLC6A5, SLC6A9, SPR, TH*	45
**19.**	**Neurodegeneration with Brain Iron Accumulation Panel**	*AP4M1, ATP13A2, C19orf12, COASY, CP, CRAT, DCAF17, FA2H, FTL, FUCA1, GJA1, GTPBP2, KIF1A, PANK2, PLA2G6, REPS1, SCP2, SLC25A42, SQSTM1, WDR45*	20
**RETINAL DISORDERS**			
**20.**	**Invitae Inherited Retinal Disorders Panel**	*ABCA4, ABCC6, ABHD12, ACBD5, ACO2, ADAM9, ADAMTS18, ADAMTSL4, ADGRA3, ADGRV1, ADIPOR1, AGBL5, AHI1, AHR, AIPL1, ALMS1, ARHGEF18, ARL13B, ARL2BP, ARL3, ARL6, ARMC9, ARSG, ASRGL1, ATF6, ATOH7, B9D1, BBIP1, BBS1, BBS10, BBS12, BBS2, BBS4, BBS5, BBS7, BBS9, BEST1, C10orf11, C12orf65, C1QTNF5,*	330

		C8orf37, CA4, CABP4, CACNA1F, CACNA2D4, CAPN5, CC2D2A, CCT2, CDH23, CDH3, CDHR1, CEP164, CEP19, CEP250, CEP290, CEP41, CEP78, CEP83, CERKL, CFAP410, CHM, CIB2, CISD2, CLCC1, CLN2 (TPP1),CLN3, CLN5, CLN6, CLN8, CLRN1, CLUAP1, CNGA1, CNGA3, CNGB1, CNGB3, CNNM4, COL11A1, COL11A2, COL18A1, COL2A1, COL9A1, COL9A2, COL9A3, CPLANE1, CRB1, CRX, CSPP1, CTNNA1, CTSD, CWC27, CYP4V2, DHDDS, DHX32, DHX38, DNAJC17, DRAM2, DSCAML1, DTHD1, EFEMP1, ELOVL4, EMC1, ERCC6, EXOSC2, EYS, FAM161A, FBLN5, FLVCR1, FRMD7, FSCN2, FZD4, GDF6, GNAT1, GNAT2, GNB3, GNPTG, GNS, GPR143, GPR179, GPR45, GRM6, GRN, GUCA1A, GUCA1B, GUCY2D, HARS, HGSNAT, HK1, HMCN1, HMX1, IDH3A, IDH3B, IFT140, IFT172, IFT27, IFT43, IFT74, IFT80, IFT81, IFT88, IMPDH1, IMPG1, IMPG2, INPP5E, INVS, IQCB1, ITM2B, JAG1, KCNJ13, KCNV2, KIAA0586, KIAA1549, KIF11, KIF7, KIZ, KLHL7, LCA5, LRAT, LRIT3, LRP2, LRP5, LYST, LZTFL1, MAK, MAPKAPK3, MERTK, MFN2, MFRP, MFSD8, MIR204, MKKS, MKS1, MPDZ, MTPAP, MTTP, MYO7A, NAGLU, NBAS, NDP, NEK2, NEUROD1, NMNAT1, NPHP1, NPHP3, NPHP4, NR2E3, NR2F1, NRL, NYX, OAT, OCA2, OFD1, OPA1, OPA3, OPN1SW, OR2W3, OTX2, P3H2, PAX2, PAX6, PCARE, PCDH15, PCYT1A, PDE6A, PDE6B, PDE6C, PDE6D, PDE6G, PDE6H, PDZD7, PEX1, PEX10, PEX11B, PEX12, PEX13, PEX14, PEX16, PEX19, PEX2, PEX26, PEX3, PEX5, PEX6, PEX7, PHYH, PITPNM3, PLA2G5, PLK4, PNPLA6, POC1B, POC5, POMGNT1, PPT1, PRCD, PRDM13, PROM1, PRPF3, PRPF31, PRPF4, PRPF6, PRPF8, PRPH2, PRPS1, RAB28, RAX2, RBP1, RBP3, RBP4, RCBTB1, RD3, RDH11, RDH12, RDH5, REEP6, RGR, RGS9, RGS9BP, RHO, RIMS1, RLBP1, ROM1, RP1, RP1L1, RP2, RP9, RPE65, RPGR, RPGR (ORF15),RPGRIP1, RPGRIP1L, RS1, RTN4IP1, SAG, SAMD11, SCLT1, SDCCAG8, SEMA4A, SGSH, SIX6, SLC24A1, SLC24A5, SLC45A2, SLC7A14, SNRNP200, SPATA7, SPP2, TCTN1, TCTN2, TCTN3, TEAD1, TIMM8A, TIMP3, TMED7, TMEM107, TMEM126A, TMEM138, TMEM216, TMEM231, TMEM237, TMEM67, TOPORS, TRAF3IP1, TREX1, TRIM32, TRNT1, TRPM1, TSPAN12, TTC21B, TTC8, TTLL5, TTPA, TUB, TUBGCP4, TUBGCP6, TULP1, TYR, TYRP1, UNC119, USH1C, USH1G, USH2A, VCAN, VPS13B, WDPCP, WDR19, WDR34, WFS1, WHRN, ZNF408, ZNF423, ZNF513	
**SINGLE GENES**			
**21.**	**Neurofibromatosis type 2**	*NF2*	1
**22.**	**TREX1**	*TREX1*	1
