# Supplementary material for: The utility of next generation sequencing targeted multigene panels in the Adult Neurogenetic Clinic at Tygerberg Hospital, South Africa
Source: Eur J Hum Genet. 2025 Jun 25;33(9):1144–52. doi: 10.1038/s41431-025-01900-2 (PMC12402217; doi:10.1038/s41431-025-01900-2)
Supplement: Supplementary file 3 — Supplementary Table 3 [file 41431_2025_1900_MOESM3_ESM.pdf]

NR	PHENOTYPIC FEATURES	GENE & VARIANT	OMIM	INHERITANCE/ ZYGOSITY	ACMG CLASSIFICATION	ACMG 2015 CRITERIA
**18**	Ataxia, spasticity, and cognitive deterioration. Brain MRI revealed significant and diffuse white matter loss in the cerebral hemispheres.	*EIF2B5*<br>c.1654+1G>T (Splice donor)<br>SCV002317851	# 620315	Autosomal Recessive<br>Heterozygous	Likely Pathogenic	PVS1<br>PM2<br>PP5
		EIF2B5<br>c.383A>G (p.Tyr128Cys)<br>SCV002213992		Heterozygous	VUS	PM2<br>PP3<br>PP2
**19**	Slowly progressive spastic paraparesis. An asymptomatic parent was found to be a heterozygous carrier for the same variant.	*CYP7B1*<br>c.1507A>G (p.Lys503Glu)<br>SCV002205090.2	# 270800	Autosomal Recessive<br>Homozygous	VUS	PM2
